# Use of Mechanical Cardiopulmonary Resuscitation Devices for Out-of-Hospital Cardiac Arrest, 2010-2016

**DOI:** 10.1001/jamanetworkopen.2019.13298

**Published:** 2019-10-16

**Authors:** Peter A. Kahn, Sanket S. Dhruva, Taeho Greg Rhee, Joseph S. Ross

**Affiliations:** 1 Department of Internal Medicine, Yale University School of Medicine, New Haven, Connecticut; 2 Section of Cardiology, Department of Medicine, University of California, San Francisco School of Medicine, San Francisco; 3 Department of Medicine, San Francisco Veterans Affairs Health Care System, San Francisco, California; 4 Department of Community Medicine and Health Care, School of Medicine, University of Connecticut, Farmington; 5 Section of General Medicine, Department of Internal Medicine, Yale University School of Medicine, New Haven, Connecticut; 6 Department of Health Policy and Management, Yale University School of Public Health, New Haven, Connecticut; 7 Center for Outcomes Research and Evaluation, Yale New Haven Health System, New Haven, Connecticut

## Abstract

**Importance:**

Out-of-hospital cardiac arrest is a common scenario facing prehospital emergency medical services (EMS) professionals and nearly always involves either manual or mechanical cardiopulmonary resuscitation (CPR). Mechanical CPR devices are expensive and prior clinical trials have not provided evidence of benefit for patients when compared with manual CPR.

**Objectives:**

To investigate the use of mechanical CPR in the prehospital setting and determine whether patient demographic characteristics or geographical location is associated with its use.

**Design, Setting, and Participants:**

A retrospective cross-sectional study was performed using the 2010 through 2016 National Emergency Medical Services Information System data. Participants included all patients identified by EMS professionals as having out-of-hospital cardiac arrest.

**Main Outcomes and Measures:**

Use of CPR, categorized as manual or mechanical.

**Results:**

From 2010 to 2016, 892 022 patients (38.6% female, 60.4% male, missing for 1%; mean [SD] age, 61.1 [20.5] years) with out-of-hospital cardiac arrest were identified by EMS professionals. Overall, manual CPR was used for 618 171 patients (69.3%) and mechanical CPR was used for 45 493 patients (5.1%). The risk-standardized rate of mechanical CPR use, accounting for patient demographic and geographical characteristics, rose from 1.9% in 2010 to 8.0% in 2016 (*P* < .001). In multivariable analyses, use of mechanical CPR devices was increasingly likely over time among patients identified with out-of-hospital cardiac arrest treated by EMS professionals, increasing from an adjusted odds ratio of 1.58 (95% CI, 1.42-1.77; *P* < .001) when comparing 2011 with 2010, to an adjusted odds ratio of 11.32 (95% CI, 10.22-12.54; *P* < .001) when comparing 2016 with 2010. In addition, several other patient demographic and geographical characteristics were associated with a higher likelihood of receiving mechanical CPR, including being 65 years or older, being male, being Hispanic, as well as receiving treatment in the Northeast Census Region, in a suburban location, or in a zip code with a median annual income greater than $20 000.

**Conclusions and Relevance:**

Mechanical CPR device use increased more than 4-fold among patients with out-of-hospital cardiac arrest treated by EMS professionals. Given the high costs of mechanical CPR devices, better evidence is needed to determine whether these devices improve clinically meaningful outcomes for patients treated for out-of-hospital cardiac arrest by prehospital EMS professionals to justify the significant increase in their use.

## Key Points

**Question:**

How frequently were mechanical cardiopulmonary resuscitation devices used in the United States by prehospital emergency medical services professionals for patients with out-of-hospital cardiac arrest from 2010 to 2016?

**Findings:**

In this cross-sectional study, the risk-adjusted use of mechanical cardiopulmonary resuscitation devices rose from 1.9% to 8.0% among all out-of-hospital cardiac arrests. This increase was consistent among patients when stratified by demographic characteristics and geographical location.

**Meaning:**

Use of mechanical cardiopulmonary resuscitation devices has increased more than 4-fold among patients with out-of-hospital cardiac arrest treated by emergency medical services professionals, despite significant costs and minimal evidence that these devices improve clinically meaningful outcomes among patients.

## Teaser

This cross-sectional study uses data from the National Emergency Medical Services Information System to investigate the use of mechanical cardiopulmonary resuscitation (CPR) in the prehospital setting and determine whether patient demographics or geographical location is associated with its use.

## Introduction

Mechanical cardiopulmonary resuscitation (CPR) devices came to market in the mid-1960s as a tool to provide consistent chest compressions during treatment for cardiac arrest. Although initial mechanical CPR devices were often cumbersome, later generations of devices slowly became portable and suitable for use outside of hospital settings. With this evolution, between 1976 and 2018, approximately 40 models of mechanical CPR devices were cleared for use by the US Food and Drug Administration through the 510(k) premarket notification pathway.

Despite the proliferation of these devices over time, there remains limited evidence supporting their use during out-of-hospital cardiac arrest. Randomized clinical trials focused on clinical outcomes have yet to demonstrate benefit when compared with manual CPR.^1,2,3,4^ Neither the PARAMEDIC (Prehospital Randomized Assessment of Mechanical Compression Device in Cardiac Arrest)^4^ trial nor the CIRC (Circulation Improving Resuscitation Care)^2^ trial, each testing different manual CPR devices among thousands of patients, found any significant differences in survival to discharge or 30-day survival between patients who received mechanical CPR and manual CPR. Furthermore, a systematic review and meta-analysis found similar results, determining there to be no significant difference in outcomes among patients with out-of-hospital cardiac arrest between those who received mechanical CPR and those who received manual CPR.^5^ Using the PARAMEDIC data, investigators also concluded that mechanical CPR was not cost-effective, demonstrating that those treated with the LUCAS 2 (Lund University Cardiopulmonary Assist System 2) mechanical CPR device experienced poorer health outcomes as measured by lower quality-adjusted life-years as well as higher health and social costs compared with those treated with manual CPR.^6^ However, data from a statewide, prospectively collected cardiac arrest registry did provide some suggestion of clinical benefit associated with mechanical CPR devices.^7^

Despite the lack of evidence that mechanical CPR devices improve clinically meaningful outcomes among patients, there are anecdotal reports suggesting that their use is increasing among emergency medical services (EMS) professionals for patients with out-of-hospital cardiac arrest.^8^ If true, such an increase would be particularly worrisome not only because of the uncertainty of clinical benefit associated with device use, but also because of the devices’ known significant costs; mechanical CPR devices are often priced higher than $10 000 per unit. Accordingly, our objectives were to characterize recent trends in mechanical CPR device use for patients identified with out-of-hospital cardiac arrest treated by prehospital EMS professionals and to determine whether patient characteristics and geographical location were associated with use.

## Methods

### Data Source

We used data from 2010 through 2016 from the National EMS Information System (NEMSIS), which aggregates information from participating emergency medical systems across the United States.^9^ NEMSIS is a voluntary national registry of EMS activations funded by the National Highway Traffic Safety Administration, and participation in NEMSIS has been increasing over time as more states and localities contribute data; in 2010, there were 3883 unique agencies contributing data, and in 2016, there were 7764 unique agencies contributing data. NEMSIS provides a database of standardized EMS patient care reports, including demographics, clinical details, procedures, medications, dispatch data, and transportation information. NEMSIS data are obtained from local EMS agencies through each reporting state and are deidentified to produce a public use data set. Because this study used public use data files, it was exempt from institutional review board approval. Our study did not require informed consent, given that our data do not contain any identifiable information and study participants cannot be contacted. This study followed the Strengthening the Reporting of Observational Studies in Epidemiology (STROBE) reporting guideline.

As of 2015, NEMSIS data included 2497 of the 3144 counties and county equivalents (79.4%) in the United States; there are no specific criteria associated with NEMSIS participation. NEMSIS seeks to develop a complete census of all ground response EMS activations occurring in the United States. Air medical transports, critical care transports, interfacility transports, and community paramedicine visits are less likely to be included in the NEMSIS data set. Nevertheless, most states require (or expect) all EMS-related activations to be included in NEMSIS. Agencies submitting databases on the NEMSIS standard, which requires that records be collected electronically and submitted using national standards, are likely to submit 100% of records using the same approach. The EMS software commonly uses data validation rules when EMS professionals are completing a patient care report. Once records are submitted to the national registry, audit filters assess more than 400 validation rules and send a data quality report back to the EMS state offices. Additional information regarding the background of NEMSIS and the agencies submitting data are available from the Technical Assistance Center and from other studies that have used NEMSIS data for research.^9,10,11^

### Study Population

We limited our sample to patients who received treatment in the field with cardiac arrest identified by prehospital EMS professionals; patients found dead at the scene, who refused care, or for whom treatment was not required were excluded. Patients with cardiac arrest were identified using information provided by EMS professionals, including a primary or secondary “clinical impression” that the patient was experiencing cardiac arrest or that a cardiac arrest protocol was initiated. All other patients, regardless of whether manual or mechanical CPR was performed, were excluded from our study.

### Main Outcome

We determined use of CPR, categorized as either manual or mechanical, based on a combination of procedure codes, including both a CPR “flag” and CPR type listed under procedure. All of the following codes were used to categorize patients as receiving mechanical CPR devices: CPR–AutoPulse device, CPR–mechanical thumper type device, or CPR by other external automated device. Manual CPR was defined based on the following categories: CPR–start compressions and ventilations, CPR–start compressions only without ventilation, CPR–start rescue breathing without compressions, or CPR–stop. Patients for whom mechanical or manual CPR was not explicitly noted were considered not to have received CPR.

Our main outcome was the proportion of out-of-hospital patients with cardiac arrest who received CPR from a mechanical CPR device. We chose to focus on proportions, instead of absolute numbers, to account for the increasing number of states and localities contributing data to NEMSIS during the study period. We also characterized patient disposition (eg, the proportion of patients who died prior to hospital transfer and the proportion who were transferred to the hospital) and the duration required for transfer, which was reported in minutes. Disposition data are provided by NEMSIS as transportation to hospital, deceased on arrival, transportation to other places (ie, home, nursing home, medical office or clinic, or police station or jail), or missing for cases for which information was not available.

### Covariates of Interest

For each patient identified with cardiac arrest by EMS professionals, we abstracted the following demographic information: age, sex, race/ethnicity, and income, as well as Census Division and urbanicity of the area where care was provided. Age was categorized as 18 years or younger, 19 to 64 years, and 65 years or older. Sex and race/ethnicity are reported by NEMSIS based on field data reported by prehospital EMS professionals. Sex was categorized as male or female and race/ethnicity was categorized as non-Hispanic white, non-Hispanic black, Hispanic, non-Hispanic other, or not recorded or refused. Income was provided by NEMSIS based on incident location zip code, which was linked to 2005-2009 US Census data to provide median zip code–based annual per capita income in 2009 inflation-adjusted dollars. In addition, EMS agencies were categorized by type as fire department, private agency, government agency other than fire department, hospital, or nonprofit organization. Finally, geographical characteristics were ascertained, including the place where the cardiac arrest occurred, categorized as home or residence, health care facility, street or highway, or other; urbanicity based on incident location, categorized as urban, suburban, rural, or wilderness; and geographical area, categorized by Census Division based on information provided to NEMSIS by the submitting EMS agency.

### Statistical Analysis

We used descriptive statistics to characterize the sample of patients identified with out-of-hospital cardiac arrest by EMS professionals, overall and for patients who received CPR from a mechanical CPR device, including transportation time and location to which patients were transported for further care. χ^2^ Tests were used to compare the demographic and geographical characteristics over time of the population of patients identified with out-of-hospital cardiac arrest and for the subgroup receiving CPR from mechanical CPR devices. We conducted multivariable logistic regression analyses that corrected for nonindependence of observations within EMS agencies (ie, clustering) using generalized estimating equations and adjusted for patient demographic and geographical characteristics, examining the association between time (categorized as the year when care occurred) and likelihood of receiving CPR from a mechanical CPR device. As a sensitivity analysis, we repeated analyses describing the demographic and geographical characteristics of patients identified with out-of-hospital cardiac arrest and rates of mechanical CPR device use, restricting analyses only to EMS agencies submitting data for all years from 2010 to 2016. Data were analyzed using Stata, version 15.1 MP/6-Core statistical software (StataCorp). All *P* values were from 2-sided tests, and results were deemed statistically significant at *P* < .05.

## Results

From 2010 through 2016, 892 022 patients (38.6% female; mean [SD] age, 61.1 [20.5] years) were identified with out-of-hospital cardiac arrest who were treated by EMS professionals, increasing from 62 840 patients in 2010 identified by 2965 agencies to 162 411 patients in 2016 identified by 5839 agencies, reflecting the increasing number states and localities contributing data to NEMSIS. Patients were most commonly 65 years or older (47.6%), men (60.4%), non-Hispanic white (45.2%), and first treated in a zip code with a median annual income of $20 000 to $29 999 (63.0%) (Table 1). The most common EMS agency type providing care was fire departments (32.5%), while the most common place where care occurred was in the patient’s home or residence (58.1%), in urban areas (76.8%), and in the Northeast Census Region (42.0%). During the sample period, the population of patients with out-of-hospital cardiac arrest was increasingly older (≥65 years: 2010, 46.1%; 2016, 47.3%; *P* < .001), was treated in a zip code with a median annual income of $30 000 or more (2010, 16.2%; 2016, 24.1%; *P* < .001), and had EMS care provided by fire departments (2010, 30.7%; 2016, 34.8%; *P* < .001), in urban areas (2010, 72.6%; 2016, 78.8%; *P* < .001), and in the Middle Atlantic Census Division (2010, 7.9%; 2016, 19.0%; *P* < .001) (eTable 1 in the Supplement).

**Table 1.** Demographic and Geographical Characteristics of Patients Who Experienced Out-of-Hospital Cardiac Arrest Identified by Prehospital EMS Professionals, 2010-2016, Overall and Stratified by Use of CPR

Characteristic	% of Patients				*P* Value^a^
	Total (N = 892 022)	Type of CPR			
		None (n = 228 620)	Manual (n = 617 931)	Mechanical (n = 45 471)	
EMS agencies, No.	8416	475	5357	2584	
Age, y^b^					
≤18	4.1	4.4	4.2	1.2	<.001
19-64	47.1	47.5	46.9	47.5	
≥65	47.6	45.9	48.0	50.6	
Sex^b^					
Male	60.4	57.1	61.5	62.9	<.001
Female	38.6	41.0	37.9	36.7	
Race/ethnicity					
Non-Hispanic white	45.2	43.1	45.8	47.0	<.001
Non-Hispanic black	13.9	12.6	14.4	13.5	
Hispanic	3.4	3.1	3.5	3.9	
Other	4.0	3.8	4.0	3.5	
No data available	33.6	37.4	32.3	32.1	
Median annual income of zip code where EMS care occurred, $^b^					
<20 000	12.0	13.6	11.7	9.5	<.001
20 000-29 999	63.0	63.0	63.3	59.0	
≥30 000	22.3	20.2	22.4	30.2	
EMS agency type providing care					
Fire department	32.5	33.9	31.6	37.1	<.001
Private, nonhospital	19.9	25.1	18.7	9.7	
Governmental, non–fire department	18.8	16.2	19.4	23.0	
Hospital	15.0	12.5	16.1	13.2	
Nonprofit organization	13.9	12.3	14.2	17.1	
Place where EMS care occurred					
Home or residence	58.1	50.5	60.6	63.4	<.001
Health care facility	11.8	19.8	9.1	8.0	
Street or highway	7.2	5.8	7.8	6.3	
Other	15.8	14.7	16.1	17.7	
No data available	7.0	9.2	6.4	4.6	
US Census Region where EMS care occurred					
Northeast	42.0	38.8	49.2	45.5	<.001
Midwest	21.5	23.3	20.8	20.8	
South	24.3	25.2	23.7	26.6	
West	12.3	12.7	12.5	7.1	
Urbanicity^b^					
Urban	76.8	77.4	76.6	75.8	<.001
Suburban	7.9	7.5	8.1	8.0	
Rural	10.1	9.5	10.3	9.3	
Wilderness	2.7	3.0	2.6	2.7	

Abbreviation: CPR, cardiopulmonary resuscitation; EMS, emergency medical services.

^a^ Representing results from tests to examine associations between patient demographic and geographical characteristics and CPR type.

^b^ Less than 3% missing data across all years.

Among all patients identified with out-of-hospital cardiac arrest treated by EMS professionals during the study period, manual CPR was used for 618 171 patients (69.3%) and mechanical CPR was used for 45 493 patients (5.1%). The risk-standardized rate of mechanical CPR use, accounting for patient demographic and geographical characteristics, increased from 1.9% of cardiac arrests in 2010 to 8.0% in 2016, a more than 4-fold increase (*P* < .001) (Figure). Overall, 85.2% of patients were transferred by EMS to a hospital for further care and 0.6% died prior to transfer; among transported patients, the median transport time was 9.0 minutes (interquartile range, 5.0-15.0 minutes) (Table 2). When stratifying analyses by receipt of manual or mechanical CPR, we found that 87.6% of patients receiving manual CPR were transferred by EMS to a hospital for further care and 0.4% died prior to transfer, whereas 91.3% of patients receiving mechanical CPR were transferred by EMS to a hospital for further care and 0.3% died prior to transfer; among patients receiving mechanical CPR, the proportion transported to a hospital increased statistically, but only slightly, from 90.5% in 2010 to 91.8% in 2016 (*P* < .001) (eTable 2 in the Supplement). We conducted sensitivity analyses among only the EMS agencies submitting data for all years from 2010 to 2016, which demonstrated similar findings with respect to overall trends in the demographic and geographic characteristics of patients identified with out-of-hospital cardiac arrest and in rates of mechanical CPR device use (eTable 3 and eTable 4 in the Supplement).

**Figure.**
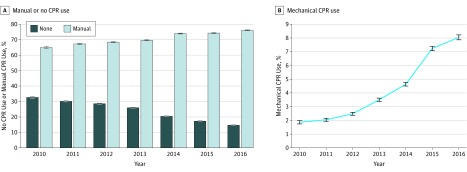
Use of Cardiopulmonary Resuscitation (CPR), Including Manual CPR and Mechanical CPR Devices, Among Patients Identified With Out-of-Hospital Cardiac Arrest Treated by Emergency Medical Services Professionals, 2010-2016 Estimates are risk standardized to account for all patient demographic and geographical characteristics listed in Table 1. Whiskers on each point estimate represent 95% CIs.

**Table 2.** Clinical Care Characteristics of Patients Who Experienced Out-of-Hospital Cardiac Arrest Identified by Prehospital EMS Professionals, 2010-2016, Overall and Stratified by Use of CPR

Characteristic	Total (N = 892 022)	CPR Type			*P* Value^a^
		None (n = 228 620)	Manual (n = 617 931)	Mechanical (n = 45 471)	
EMS agencies, No.	8416	475	5357	2584	
Patient disposition, %					
Transferred to hospital	85.2	77.5	87.6	91.3	<.001
Died	0.6	1.45	0.4	0.3	
Transferred to other places	2.1	4.86	1.1	0.8	
No data available	12.1	16.2	11.0	7.7	
Transport time, median (IQR), min	9.0 (5.0-15.0)	10.8 (6.0-18.0)	8.3 (5.0-13.7)	9.0 (5.6-13.7)	.94

Abbreviations: CPR, cardiopulmonary resuscitation; EMS, emergency medical services; IQR, interquartile range.

^a^ Representing results from tests to examine associations between patient clinical care characteristics and CPR type.

The number of EMS agencies reporting any use of mechanical CPR devices for patients identified with out-of-hospital cardiac arrest increased from 198 in 2010 (6.7% of agencies reporting to NEMSIS) to 1428 in 2016 (24.5% of agencies) (*P* < .001) (eTable 5 in the Supplement). Characteristics of patients receiving mechanical CPR were fairly consistent over time, with small observed increases in the proportion of patients who were non-Hispanic, were treated in a zip code with a median annual income between $20 000 and $29 999, and had EMS care provided by fire departments, in suburban areas, and in the East North Central and South Atlantic Census Divisions (*P* < .001) (eTable 5 in the Supplement).

In multivariable analyses, use of mechanical CPR devices was increasingly likely over time among patients identified with out-of-hospital cardiac arrest treated by EMS professionals, increasing from an adjusted odds ratio of 1.58 (95% CI, 1.42-1.77; *P* < .001) when comparing 2011 with 2010, to an adjusted odds ratio of 11.32 (95% CI, 10.22-12.54; *P* < .001) when comparing 2016 with 2010 (Table 3). In addition, several other patient demographic and geographical characteristics were associated with a higher likelihood of receiving CPR with a mechanical device: being 65 years or older, being male, being Hispanic, as well as receiving treatment in the Northeast Census Region, a suburban location, or a zip code with a median annual income greater than $20 000 (Table 3), in addition to there being differences by EMS agency type.

**Table 3.** Likelihood of Receiving Mechanical Cardiopulmonary Resuscitation Among Patients Who Experienced Out-of-Hospital Cardiac Arrest Identified by Prehospital EMS Professionals

Characteristic	Bivariate		Adjusted^a^	
	OR (95% CI)	*P* Value	OR (95% CI)	*P* Value
**Year**				
2010	1 [Reference]	NA	1 [Reference]	NA
2011	1.57 (1.42-1.74)	<.001	1.58 (1.42-1.77)	<.001
2012	2.31 (2.10-2.55)	<.001	2.35 (2.11-2.62)	<.001
2013	3.32 (3.02-3.64)	<.001	3.32 (2.99-3.68)	<.001
2014	5.15 (4.69-5.65)	<.001	5.24 (4.73-5.80)	<.001
2015	7.61 (6.94-8.35)	<.001	7.95 (7.18-8.80)	<.001
2016	10.45 (9.53-11.46)	<.001	11.32 (10.22-12.54)	<.001
Age, y				
≤18	0.23 (0.21-0.26)	<.001	0.20 (0.18-0.23)	<.001
19-64	0.96 (0.94-0.99)	.004	0.92 (0.89-0.95)	<.001
≥65	1 [Reference]	NA	1 [Reference]	NA
Sex				
Male	1 [Reference]	NA	1 [Reference]	NA
Female	0.89 (0.87-0.92)	<.001	0.90 (0.88-0.93)	<.001
Race/ethnicity				
Non-Hispanic white	1 [Reference]	NA	1 [Reference]	NA
Non-Hispanic black	0.97 (0.92-1.02)	.22	0.97 (0.92-1.03)	.37
Hispanic	1.13 (1.04-1.23)	.004	1.16 (1.06-1.28)	.002
Other	0.97 (0.89-1.05)	.46	1.02 (0.93-1.12)	.71
No data available	1.02 (0.98-1.05)	.40	1.00 (0.96-1.04)	.92
Median annual income of zip code where EMS care occurred, $				
<20 000	1 [Reference]	NA	1 [Reference]	NA
20 000-29 999	1.39 (1.29-1.50)	<.001	1.11 (1.01-1.22)	.03
≥30 000	2.16 (1.96-2.39)	<.001	1.64 (1.45-1.86)	<.001
EMS agency type providing care				
Fire department	1 [Reference]	NA	1 [Reference]	NA
Private, nonhospital	0.31 (0.28-0.34)	<.001	0.32 (0.28-0.36)	<.001
Governmental, non–fire department	0.77 (0.71-0.85)	<.001	0.83 (0.74-0.92)	.001
Hospital	0.77 (0.70-0.85)	<.001	0.92 (0.82-1.04)	.17
Nonprofit organization	1.02 (0.94-1.10)	.67	0.98 (0.89-1.08)	.75
Transport time in min	1.00 (1.00-1.00)	.68	1.00 (1.00-1.00)	.68
Place where EMS care occurred				
Home or residence	1 [Reference]	NA	1 [Reference]	NA
Health care facility	0.64 (0.61-0.68)	<.001	0.60 (0.57-0.63)	<.001
Street or highway	0.82 (0.77-0.86)	<.001	0.80 (0.75-0.85)	<.001
Other	0.95 (0.91-0.98)	.005	0.88 (0.85-0.92)	<.001
US Census Region				
Northeast	1 [Reference]	NA	1 [Reference]	NA
Midwest	0.55 (0.50-0.60)	<.001	0.72 (0.64-0.80)	<.001
South	0.42 (0.38-0.46)	<.001	0.42 (0.38-0.47)	<.001
West	0.70 (0.62-0.79)	<.001	0.78 (0.68-0.90)	.001
Urbanicity				
Urban	1 [Reference]	NA	1 [Reference]	NA
Suburban	0.89 (0.81-0.97)	.01	1.37 (1.23-1.52)	<.001
Rural	0.55 (0.51-0.60)	<.001	0.66 (0.60-0.74)	<.001
Wilderness	0.85 (0.74-0.97)	.01	1.08 (0.92-1.25)	.37

Abbreviations: EMS, emergency medical services; NA, not applicable; OR, odds ratio.

^a^ Analyses adjusted for all patient and geographical characteristics listed.

## Discussion

Between 2010 and 2016, use of mechanical CPR devices for patients with out-of-hospital cardiac arrest treated by EMS professionals increased more than 4-fold, with wide variation in use across communities by EMS agency type and Census Division. The increasing use of mechanical CPR devices for out-of-hospital cardiac arrest raises concerns because of their costliness and lack of evidence demonstrating clinical benefit compared with manual CPR, as randomized clinical trials have failed to demonstrate a clinical benefit of mechanical CPR compared with manual CPR for out-of-hospital cardiac arrest.^1,2,3,4^ Furthermore, the safety of these devices, including their potential to cause serious injuries to organs, large vessels, or vertebrae as compared with manual CPR, has not been reliably evaluated in real-world use, outside of clinical trial settings, where EMS personnel may receive less training in their use and application or have less experience.

Efforts are needed to better understand why prehospital EMS professionals may prefer mechanical CPR devices, particularly given their expense. Undoubtedly, there are likely to be clinical circumstances for which these devices may be particularly useful, which could be driving use, such as when transportation times are expected to be prolonged, in underresourced settings where personnel are limited, during motor vehicle extrications after crashes, and during prehospital transfers that involve stairways or transportation over uneven ground. Similarly, the increasing use of mechanical CPR devices could be driven, in part, by 2015 CPR guidelines that emphasized compressions rather than rescue breathing.^12^ Further research is needed to better understand the utility of these devices for EMS professionals in these settings, or whether there are other benefits of mechanical CPR devices to patient care that have yet to be described, including EMS professional satisfaction and workload.

### Limitations

There are several important limitations to this study. First, our study demonstrates the increasing use of mechanical CPR devices for out-of-hospital cardiac arrest treated by EMS professionals. Because there was an increasing number of states and localities contributing data to NEMSIS, we cannot be certain that our findings are not a consequence of the changing patient population, as opposed to changing use of these new medical devices. However, our sensitivity analyses limited to agencies reporting data to NEMSIS in each year of our study suggest that the changing patient population is unlikely to account for our findings. Furthermore, the broad consistencies in the demographic and geographical characteristics of patients identified with out-of-hospital cardiac arrest, along with the substantial increase in the use of mechanical CPR devices and the stable delivery of manual CPR, suggest that these trends in the use of mechanical CPR devices are real.

Second, our study used data from NEMSIS, the best available information on the care delivered in the community by prehospital EMS professionals. Although NEMSIS is a national database including data from most states, some states and some agencies within reporting states do not submit data. Moreover, NEMSIS lacks information on the clinical appropriateness of mechanical CPR device use, actual costs, and number of unique professionals who have used the device, as well as granular information on patient outcomes after use of a mechanical CPR device. Third, our study does not account for the use of mechanical CPR devices by prehospital EMS professionals treating patients who are not experiencing out-of-hospital cardiac arrest, nor does it account for use in hospitals, emergency departments, or other urgent care facilities.

## Conclusions

Between 2010 and 2016, use of mechanical CPR devices for patients identified with out-of-hospital cardiac arrest treated by EMS professionals increased more than 4-fold, with wide variation in use across communities by agency type and Census Division. Our findings should be used to inform current state and local EMS efforts to use cost-effective, high-quality care for patients experiencing cardiac arrest in the community. Although mechanical CPR devices may be useful in certain clinical circumstances, the routine and increasing use of mechanical CPR devices for out-of-hospital cardiac arrest does not appear to be justified by the current available evidence. To justify the significant increase in use of these devices, better evidence is needed of improved, clinically meaningful outcomes for patients with out-of-hospital cardiac arrest treated with mechanical CPR by prehospital EMS professionals.

## Notes

Supplement. **eTable 1.** Demographic and Geographic Characteristics of Patients Who Experienced Out-of-Hospital Cardiac Arrest Identified by Prehospital Emergency Medical Services (EMS) Professionals, 2010-2016 **eTable 2.** Clinical Care Characteristics of Patients Who Experienced Out-of-Hospital Cardiac Arrest Identified by Prehospital Emergency Medical Services (EMS) Professionals, 2010-2016 **eTable 3.** Demographic and Geographic Characteristics of Patients Who Experienced Out-of-Hospital Cardiac Arrest Identified by Prehospital Emergency Medical Services (EMS) Professionals Who Reported Data to NEMSIS in Each Sample Year, 2010-2016 **eTable 4.** Clinical Care Characteristics of Patients Who Experienced Out-of-Hospital Cardiac Arrest Identified by Prehospital Emergency Medical Services (EMS) Professionals Who Reported Data to NEMSIS in Each Sample Year, 2010-2016 **eTable 5.** Demographic and Geographic Characteristics of Patients Who Experienced Out-of-Hospital Cardiac Arrest Identified by Prehospital EMS Professionals and Received Mechanical CPR Devices, 2010-2016
