# Supplementary material for: Use of Mechanical Cardiopulmonary Resuscitation Devices for Out-of-Hospital Cardiac Arrest, 2010-2016
Source: JAMA Netw Open. 2019 Oct 16;2(10):e1913298. doi: 10.1001/jamanetworkopen.2019.13298 (PMC6806423; doi:10.1001/jamanetworkopen.2019.13298)
Supplement: Supplement. — eTable 1. Demographic and Geographic Characteristics of Patients Who Experienced Out-of-Hospital Cardiac Arrest Identified by Prehospital Emergency Medical Services (EMS) Professionals, 2010-2016 eTable 2. Clinical Care Characteristics of Patients Who Experienced Out-of-Hospital Cardiac Arrest Identified by Prehospital Emergency Medical Services (EMS) Professionals, 2010-2016 eTable 3. Demographic and Geographic Characteristics of Patients Who Experienced Out-of-Hospital Cardiac Arrest Identified by Prehospital Emergency Medical Services (EMS) Professionals Who Reported Data to NEMSIS in Each Sample Year, 2010-2016 eTable 4. Clinical Care Characteristics of Patients Who Experienced Out-of-Hospital Cardiac Arrest Identified by Prehospital Emergency Medical Services (EMS) Professionals Who Reported Data to NEMSIS in Each Sample Year, 2010-2016 eTable 5. Demographic and Geographic Characteristics of Patients Who Experienced Out-of-Hospital Cardiac Arrest Identified by Prehospital EMS Professionals and Received Mechanical CPR Devices, 2010-2016 [file jamanetwopen-2-e1913298-s001.pdf]

Supplementary Online Content

Kahn PA, Dhruva SS, Rhee TG, Ross JS. Use of mechanical cardiopulmonary resuscitation devices for out-of-hospital cardiac arrest, 2010-2016. *JAMA Netw Open.* 2019;2(10):e1913298. doi:10.1001/jamanetworkopen.2019.13298

eTable 1. Demographic and Geographic Characteristics of Patients Who Experienced Out-of-Hospital Cardiac Arrest Identified by Prehospital Emergency Medical Services (EMS) Professionals, 2010-2016
eTable 2. Clinical Care Characteristics of Patients Who Experienced Out-of-Hospital Cardiac Arrest Identified by Prehospital Emergency Medical Services (EMS) Professionals, 2010-2016
eTable 3. Demographic and Geographic Characteristics of Patients Who Experienced Out-of-Hospital Cardiac Arrest Identified by Prehospital Emergency Medical Services (EMS) Professionals Who Reported Data to NEMSIS in Each Sample Year, 2010-2016
eTable 4. Clinical Care Characteristics of Patients Who Experienced Out-of-Hospital Cardiac Arrest Identified by Prehospital Emergency Medical Services (EMS) Professionals Who Reported Data to NEMSIS in Each Sample Year, 2010-2016
eTable 5. Demographic and Geographic Characteristics of Patients Who Experienced Out-of-Hospital Cardiac Arrest Identified by Prehospital EMS Professionals and Received Mechanical CPR Devices, 2010-2016

This supplementary material has been provided by the authors to give readers additional information about their work.

© 2019 Kahn PA et al. *JAMA Network Open.*

eTable 1. Demographic and Geographic Characteristics of Patients Who Experienced Out-of-Hospital Cardiac Arrest Identified by Prehospital Emergency Medical Services (EMS) Professionals, 2010-2016

	2010	2011	2012	2013	2014	2015	2016	Total
Patients, No.	62,840	92,838	123,472	137,602	145,425	167,434	162,411	892,022
EMS Agencies, No.	2,965	4,185	5,228	5,670	5,869	6,058	5,839	8,416
Age								
0-18	4.9	4.5	4.2	3.9	3.9	4.0	4.0	4.1
19-64	47.0	47.6	47.1	46.5	46.4	47.2	47.6	47.1
≥65	46.1	46.8	47.5	48.4	48.5	47.5	47.3	47.6
Sex								
Male	60.1	59.6	59.7	60.3	60.7	60.8	61.1	60.4
Female	38.9	39.6	39.4	38.7	38.3	38.2	38.0	38.6
Race/ethnicity								
Non-Hispanic white	47.5	47.8	48.3	47.4	46.0	43.3	39.9	45.2
Non-Hispanic black	12.2	13.0	13.9	15.3	14.5	13.2	13.8	13.9
Hispanic	3.4	2.9	2.9	3.3	3.5	3.4	4.1	3.4
Other	4.5	3.8	4.7	4.0	3.7	3.6	3.8	4.0
No data available	32.4	32.5	30.2	30.0	32.3	36.5	38.4	33.6
Median annual income of zip code where EMS care occurred								
<$20,000	14.9	14.4	11.7	11.7	11.7	11.3	11.2	12.0
$20,000 to $29,999	60.9	62.3	63.4	63.6	63.5	63.5	62.3	63.0
≥$30,000	16.2	20.6	22.6	22.7	22.7	22.6	24.1	22.3
EMS agency type providing care								
Fire department	30.7	29.3	29.2	32.2	33.6	34.2	34.8	32.5
Private, non-hospital	17.0	19.8	20.0	19.9	19.4	20.6	20.8	19.9
Governmental, non-fire department	26.9	21.5	19.6	18.5	17.8	16.6	16.7	18.8
Hospital	16.0	15.3	14.3	12.4	16.0	15.0	16.4	15.0

© 2019 Kahn PA et al. *JAMA Network Open.*

Non-profit organization	9.5	14.1	16.9	17.0	13.3	13.5	11.3	13.9
Place where EMS care occurred								
Home or residence	57.8	57.4	57.4	59.3	59.3	58.8	56.6	58.1
Healthcare facility	14.9	12.8	13.2	12.6	11.2	11.1	9.7	11.8
Street or highway	6.4	6.3	6.9	6.7	7.4	8.0	7.6	7.2
Other	13.7	14.4	14.6	15.1	16.2	16.1	18.5	15.8
No data available	7.2	9.2	7.8	6.3	6.1	6.0	7.7	7.0
US Census Division location where EMS care occurred								
East North Central	10.4	9.7	11.7	14.1	14.0	14.3	13.6	13.0
East South Central	8.9	6.9	6.4	5.9	5.3	5.7	5.2	6.0
Middle Atlantic	7.9	13.2	17.6	19.0	20.5	19.1	19.0	17.7
Mountain	11.8	7.6	6.9	6.2	6.4	6.4	6.0	6.9
New England	2.6	2.2	3.6	3.2	4.0	4.8	4.3	3.7
Pacific	3.3	2.4	1.8	4.8	5.3	7.2	8.5	5.2
South Atlantic	37.2	40.6	36.5	36.1	33.0	30.4	33.5	34.6
Territories	0.1	0.1	0.1	0.3	0.3	0.3	0.3	0.2
West North Central	10.3	12.2	10.9	5.9	7.0	6.6	6.5	8.0
West South Central	7.7	5.2	4.4	4.5	4.4	5.4	3.3	4.7
Urbanicity of area where EMS care occurred								
Urban	72.6	74.2	76.2	77.2	76.9	77.8	78.8	76.8
Suburban	10.0	8.7	8.2	7.8	7.7	7.7	7.2	7.9
Rural	13.0	12.8	11.3	10.1	9.3	8.7	8.4	10.1
Wilderness	4.2	3.2	2.8	2.8	2.5	2.3	2.3	2.7

Note: Age, sex, income, and urbanicity had missing data less than 3% across all years.

© 2019 Kahn PA et al. *JAMA Network Open.*

eTable 2. Clinical Care Characteristics of Patients Who Experienced Out-of-Hospital Cardiac Arrest Identified by Prehospital Emergency Medical Services (EMS) Professionals, 2010-2016

	2010	2011	2012	2013	2014	2015	2016	Total
Patients, No.	62,840	92,838	123,472	137,602	145,425	167,434	162,411	892,022
EMS Agencies, No.	2,965	4,185	5,228	5,670	5,869	6,058	5,839	8,416
Received CPR								
Manual CPR only	63.2	62.4	64.8	68.4	72.5	72.4	73.6	69.3
Mechanical CPR related	2.1	2.5	3.0	3.9	5.2	6.8	8.5	5.1
None or missing	34.8	35.2	32.1	27.6	22.4	20.9	17.8	25.6
Received CPR*								
Manual CPR only	65.0	67.4	68.4	69.7	74.0	74.3	76.0	70.4
Mechanical CPR related	1.9	2.0	2.5	3.5	4.6	7.2	8.0	4.2
None or missing	32.7	30.1	28.5	25.9	20.4	17.3	14.6	24.6
Median transport time in minutes (IQR)	9.0 (5 - 15)	9.5 (5 - 16)	9.0 (5 - 15)	9.0 (5 - 14)	9.0 (5 - 14)	8.9 (5 - 14)	8.9 (5 - 14)	9.0 (5 - 15)
Disposition								
Transferred to hospital	86.5	84.4	83.7	84.9	85.7	86.0	85.1	85.2
Died	0.7	0.7	0.6	0.6	0.5	0.7	0.7	0.6
Transferred to other places	2.5	2.1	2.7	2.9	2.1	1.4	1.4	2.1
No data available	10.3	12.9	13.1	11.7	11.7	11.8	12.7	12.1
Disposition by CPR type								
Manual CPR only								
Transferred to hospital	88.4	85.9	86.3	88.6	88.2	88.2	86.8	87.6
Died	0.4	0.4	0.3	0.3	0.3	0.4	0.3	0.4
Transferred to other places	1.9	1.5	1.2	1.1	1.0	0.9	1.0	1.1
No data available	9.3	12.2	12.1	10.0	10.5	10.6	11.8	11.0
Mechanical CPR related								
Transferred to hospital	90.5	89.9	90.9	91.6	91.6	90.8	91.8	91.3
Died	0.3	0.3	0.2	0.5	0.3	0.3	0.3	0.3
Transferred to other places	0.5	0.7	1.1	1.0	0.7	0.8	0.6	0.8
No data available	8.8	9.2	7.8	6.9	7.5	8.1	7.3	7.7

Note: CPR=Cardiopulmonary resuscitation; IQR=Inter-quartile range.

© 2019 Kahn PA et al. *JAMA Network Open.*

* Estimates are risk-standardized to account for all patient demographic and geographic characteristics listed in Table 1.

© 2019 Kahn PA et al. *JAMA Network Open.*

eTable 3. Demographic and Geographic Characteristics of Patients Who Experienced Out-of-Hospital Cardiac Arrest Identified by Prehospital Emergency Medical Services (EMS) Professionals Who Reported Data to NEMSIS in Each Sample Year, 2010-2016

	2010	2011	2012	2013	2014	2015	2016	Total
Patients, No.	55,698	68,035	76,038	74,547	82,165	93,773	91,695	541,951
EMS Agencies, No.	2,905	2,905	2,905	2,905	2,905	2,905	2,905	2,905
Age								
0-18	4.9	4.4	4.3	4.0	4.1	4.1	4.0	4.2
19-64	46.9	48.0	47.4	46.9	46.7	47.2	47.4	47.2
≥65	46.3	46.5	47.1	47.9	48.1	47.7	47.6	47.4
Sex								
Male	60.3	60.1	59.8	60.6	60.3	60.6	60.7	60.4
Female	38.7	39.0	39.2	38.4	38.7	38.6	38.4	38.7
Race/ethnicity								
Non-Hispanic white	46.8	47.3	47.9	50.5	47.7	46.4	42.8	46.9
Non-Hispanic black	12.5	13.6	14.3	15.6	15.8	15.2	15.6	14.8
Hispanic	3.3	3.1	3.2	3.4	3.6	3.5	4.1	3.5
Other	4.6	3.7	4.1	3.8	3.5	3.5	3.5	3.8
No data available	32.9	32.3	30.5	26.8	29.4	31.5	33.9	31.1
Median annual income of zip code where EMS care occurred								
<$20,000	15.2	15.5	14.2	15.5	15.7	15.3	14.8	15.2
$20,000 to $29,999	61.5	64.4	64.5	64.6	63.5	63.9	63.7	63.8
≥$30,000	15.5	17.7	19.0	18.7	19.4	18.2	19.2	18.4
EMS agency type providing care								
Fire department	31.0	27.8	27.1	26.6	23.6	24.6	27.1	26.6
Private, non-hospital	14.9	19.8	20.5	20.9	20.6	22.5	20.5	20.3
Governmental, non-fire department	29.0	26.2	24.9	26.1	24.4	22.9	21.8	24.7
Hospital	15.2	17.3	18.0	16.7	22.4	22.0	23.9	19.8

© 2019 Kahn PA et al. *JAMA Network Open.*

	Non-profit organization	9.9	9.0	9.6	9.8	8.9	8.0	6.7	8.7
Place where cardiac arrest occurred									
	Home or residence	58.4	58.6	58.4	60.5	60.8	59.2	56.5	58.9
	Healthcare facility	14.9	12.4	12.3	12.5	10.8	11.3	9.8	11.8
	Street or highway	6.4	6.3	6.6	6.1	7.1	7.6	7.2	6.8
	Other	13.7	13.7	14.0	14.5	15.3	15.8	18.8	15.3
	No data available	6.6	8.9	8.8	6.5	6.1	6.1	7.7	7.2
US Census Division where EMS care occurred									
	East North Central	9.7	10.3	9.3	10.9	10.6	11.5	11.3	10.6
	East South Central	9.2	7.4	7.8	7.9	7.1	7.0	6.6	7.5
	Middle Atlantic	7.5	9.1	10.9	12.3	18.0	17.4	19.1	14.1
	Mountain	12.0	9.3	8.9	8.6	7.5	6.9	6.3	8.2
	New England	2.8	2.9	2.4	2.3	2.5	2.3	1.7	2.4
	Pacific	2.6	2.4	2.2	3.3	3.1	2.6	2.9	2.8
	South Atlantic	38.8	39.3	39.4	40.7	37.0	36.2	40.4	38.8
	Territories	0.1	0.1	0.1	0.1	0.1	0.1	0.1	0.1
	West North Central	9.5	13.0	12.6	6.6	7.6	8.1	7.4	9.1
	West South Central	7.7	6.2	6.3	7.3	6.6	8.0	4.2	6.6
Urbanicity where EMS care occurred									
	Urban	72.5	74.5	75.1	73.7	74.2	74.9	76.9	74.7
	Suburban	10.8	9.8	9.4	9.6	9.0	8.6	7.7	9.1
	Rural	12.8	12.3	12.0	12.2	10.8	10.4	9.7	11.3
	Wilderness	3.7	3.0	3.1	3.4	3.0	2.8	2.7	3.0

Note: Age, sex, income, and urbanicity had missing data less than 3% across all years.

© 2019 Kahn PA et al. *JAMA Network Open.*

eTable 4. Clinical Care Characteristics of Patients Who Experienced Out-of-Hospital Cardiac Arrest Identified by Prehospital Emergency Medical Services (EMS) Professionals Who Reported Data to NEMSIS in Each Sample Year, 2010-2016

	2010	2011	2012	2013	2014	2015	2016	Total
Patients, No.	55,698	68,035	76,038	74,547	82,165	93,773	91,695	541,951
EMS Agencies, No.	2,905	2,905	2,905	2,905	2,905	2,905	2,905	2,905
Received CPR								
Manual CPR only	64.0	63.5	63.5	66.9	72.6	73.9	75.3	69.2
Mechanical CPR related	2.1	2.4	3.4	4.3	5.4	7.6	9.3	5.3
None or missing	34.0	34.0	33.1	28.7	22.0	18.5	15.4	25.5
Median transport time in minutes (IQR)	9.0 (5.0 - 15.1)	9.0 (5.0 - 15.5)	9.0 (5.1 - 15.1)	9.0 (5.0 - 15.0)	9.0 (5.0 - 14.6)	9.0 (5.0 - 14.8)	9.0 (5.1 - 14.6)	9.0 (5.0 - 15.0)
Disposition								
Transferred to hospital	86.4	85.2	84.4	85.2	85.9	86.9	86.1	85.8
Died	0.7	0.7	0.7	0.7	0.7	0.7	0.7	0.7
Transferred to other places	2.5	2.2	1.8	1.9	1.5	1.4	1.3	1.7
No data available	10.4	11.9	13.1	12.2	11.9	11.1	11.9	11.8
Disposition by CPR type								
Manual CPR only								
Transferred to hospital	88.1	86.5	85.9	88.0	87.6	88.4	87.1	87.4
Died	0.4	0.4	0.4	0.4	0.4	0.4	0.4	0.4
Transferred to other places	2.0	1.8	1.4	1.4	1.1	0.8	1.0	1.3
No data available	9.5	11.4	12.3	10.3	10.9	10.4	11.6	11.0
Mechanical CPR related								
Transferred to hospital	90.1	91.5	90.5	89.3	90.1	90.9	91.3	90.7
Died	0.4	0.3	0.2	0.5	0.3	0.3	0.4	0.3
Transferred to other places	0.5	1.0	1.3	1.5	0.8	1.0	0.7	0.9
No data available	9.0	7.3	7.9	8.8	8.8	7.9	7.7	8.1

Note: CPR=Cardiopulmonary resuscitation; IQR=Inter-quartile range.

© 2019 Kahn PA et al. *JAMA Network Open.*

eTable 5. Demographic and Geographic Characteristics of Patients Who Experienced Out-of-Hospital Cardiac Arrest Identified by Prehospital EMS Professionals and Received Mechanical CPR Devices, 2010-2016

	2010	2011	2012	2013	2014	2015	2016	Total
Patients, No.	1,312	2,276	3,736	5,410	7,490	11,373	13,874	45,471
EMS Agencies, No.	198	399	673	889	1,095	1,322	1,428	2,584
Age								
0-18	1.3	1.7	0.9	1.2	1.1	1.2	1.3	1.2
19-64	49.1	48.5	47.3	46.6	46.8	47.6	48.0	47.5
≥65	49.0	49.2	51.4	51.5	51.3	50.5	50.1	50.6
Sex								
Male	63.0	62.8	63.8	62.5	64.0	62.3	62.6	62.9
Female	36.4	36.6	35.5	36.7	35.4	37.3	37.2	36.7
Race/ethnicity								
Non-Hispanic white	38.8	40.7	44.2	50.2	50.8	51.1	43.1	47.0
Non-Hispanic black	13.0	14.1	13.0	16.1	13.1	13.6	12.8	13.5
Hispanic	4.7	3.6	3.9	2.7	3.2	3.8	4.7	3.9
Other	4.8	3.9	4.0	2.7	3.3	3.5	3.7	3.5
No data available	38.6	37.7	35.0	28.4	29.7	28.1	35.7	32.1
Median annual income of zip code where EMS care occurred								
<$20,000	11.9	12.4	12.2	10.7	10.0	8.6	7.9	9.5
$20,000 to $29,999	50.9	61.9	56.0	58.2	56.7	61.4	59.8	59.0
≥$30,000	36.1	25.6	31.1	30.2	31.9	28.7	30.6	30.2
EMS agency type providing care								
Fire department	28.0	23.3	21.8	27.9	37.2	42.2	43.7	37.1
Private, non-hospital	16.6	15.8	11.6	9.3	7.6	8.2	10.2	9.7
Governmental, non-fire department	27.4	23.5	23.5	24.9	23.8	23.3	20.8	23.0
Hospital	22.5	20.9	22.3	15.3	14.5	9.9	9.7	13.2

© 2019 Kahn PA et al. *JAMA Network Open.*

	Non-profit organization	5.6	16.5	20.8	22.6	17.0	16.4	15.7	17.1
Place where EMS care occurred									
	Home or residence	69.1	65.8	65.4	66.3	64.8	64.6	59.1	63.4
	Healthcare facility	7.2	7.2	7.8	9.1	8.5	9.0	6.9	8.0
	Street or highway	5.3	5.7	5.2	5.1	6.6	7.0	6.6	6.3
	Other	13.6	15.1	14.2	15.6	16.5	15.8	22.5	17.7
	No data available	4.8	6.3	7.4	3.9	3.7	3.5	5.1	4.6
US Census Division where EMS care occurred									
	East North Central	2.1	5.3	7.8	7.2	8.5	8.3	8.1	7.8
	East South Central	11.8	8.9	6.1	10.1	6.1	4.0	4.9	6.0
	Middle Atlantic	19.8	23.4	24.7	21.5	14.9	10.8	9.2	14.3
	Mountain	8.6	6.0	5.0	4.4	3.6	4.0	3.7	4.2
	New England	3.3	2.4	3.2	3.6	4.3	3.2	1.9	3.0
	Pacific	4.7	3.1	1.1	2.2	3.1	2.7	3.4	2.8
	South Atlantic	21.1	31.2	30.1	40.8	43.6	51.8	55.0	46.4
	Territories	0.0	0.0	0.2	0.4	0.4	0.2	0.2	0.3
	West North Central	26.5	17.9	19.5	7.8	12.3	11.2	11.8	12.6
	West South Central	2.2	1.9	2.3	2.1	3.2	3.8	1.9	2.7
Urbanicity where EMS care occurred									
	Urban	83.0	76.6	76.3	73.4	70.9	77.7	76.8	75.8
	Suburban	4.4	5.6	8.4	9.5	9.2	8.1	7.2	8.0
	Rural	10.1	13.6	11.2	9.5	9.7	8.3	8.5	9.3
	Wilderness	2.4	3.8	3.5	3.4	3.1	2.6	2.0	2.7

Note: CPR=Cardiopulmonary resuscitation; EMS=Emergency medical services. Age, sex, income, and urbanicity had missing data less than 3% across all years.

© 2019 Kahn PA et al. *JAMA Network Open.*
